# Exploring the Association Between Electronic Wearable Device Use and Levels of Physical Activity Among Individuals With Depression and Anxiety: A Population Level Study

**DOI:** 10.3389/fdgth.2021.707900

**Published:** 2021-09-14

**Authors:** Henry Onyeaka, Joe Firth, Valentine Enemuo, Chioma Muoghalu, John Naslund, Philip Baiden, John Torous

**Affiliations:** ^1^Massachusetts General Hospital, Harvard Medical School, Boston, MA, United States; ^2^Division of Psychology and Mental Health, The University of Manchester, Manchester, United Kingdom; ^3^Nnamdi Azikiwe University, Awka, Nigeria; ^4^Monmouth Medical Center, Long Branch, NJ, United States; ^5^Department of Global Health and Social Medicine, Harvard Medical School, Boston, MA, United States; ^6^School of Social Work, University of Texas at Arlington, Arlington, TX, United States; ^7^Department of Psychiatry, Beth Israel Deaconess Medical Center, Harvard Medical School, Boston, MA, United States

**Keywords:** physical activity, electronic wearable devices, mental illness, digital tool, common mental disorder

## Abstract

**Aim:** The present study aimed to investigate the cross-sectional association between self-reported use of electronic wearable devices (EWDs) and the levels of physical activity among a representative sample of adults with depression and anxiety in the United States.

**Methods:** For this cross-sectional study, data were pooled from the Health Information National Trends Survey 2019. A sample of 1,139 adults with self-reported depression and anxiety (60.9% women; mean age of 52.5 years) was analyzed. The levels of physical activity and prevalence of EWD utilization were self-reported. The chi-square tests were used to compare individual characteristics through the use of EWDs. Multivariable logistic regression was employed to investigate the association between EWDs and physical activity levels while adjusting for sociodemographic and health-related factors.

**Results:** From the 1,139 adults with self-reported depression and anxiety, 261 (weighted percentage 28.1%) endorsed using EWD in the last year. After adjusting for covariates, the use of EWDs was only significantly associated with a higher odds of reporting intention to lose weight (OR 2.12; 95% *CI* 1.04, 4.35; *p* = 0.04). We found no association between the use of EWDs and meeting the national weekly recommendation for physical activity or resistance/strength exercise training.

**Conclusion:** About three in 10 adults suffering from depression and anxiety in the United States reported using EWDs in the last year. The current study findings indicate that among people living with mental illness, EWD use is associated with higher odds of weight loss intent suggesting that EWDs may serve as an opening for the clinical interactions around physical health through identifying patients primed for behavior change. Further large-scale studies using randomized trial designs are needed to examine the causal relationships between EWDs and the physical activity of people with mental health conditions.

## Introduction

Despite the proven benefits of physical activity on the outcomes of mental health (1, 2), people living with mental illness have higher rates of physical inactivity which contributes to elevated risk of cardiovascular diseases and associated premature mortality in these populations (3). The benefits of physical activity are broad and may include symptom reduction, improved cognition, and improved quality of life among others (4, 5). The effect size of exercise for depression has been estimated to be similar to that of common antidepressant medications (4, 6). Despite these benefits, people living with mental illness may face additional barriers toward engaging in exercise, resulting in many individuals not meeting even minimum standards for regular physical activity (2, 7).

Wearable technologies offer promise in promoting physical activity as they are inexpensive, feasible, and readily acceptable by those living with mental illness (8). However, studies on the use of electronic wearable devices (EWDs) in mental health populations have been limited by small sample sizes, restriction to hospital settings, and not being nationally representative (8–10). To guide further research on the scalability and implementation of these tools in real-world settings and to potentially serve as a supplement to routine clinical services, an understanding of the association between use of EWDs and engagement in physical activity among those with mental health conditions at the population level is a necessary first step. Therefore, this study aimed to assess the prevalence of EWDs and the association between the use of EWDs and the levels of physical activity among a representative sample of adults with depression and anxiety in the United States.

## Methods

Data for this study were extracted from cycle 3 of the fifth iteration of the Health Information National Trends Survey (HINTS 5; cycle 3), a National Cancer Institute-administered survey of adults in the United States, which is nationally representative and was conducted from January 22 to April 30, 2019. The full sampling and weighting process of the H5c3 dataset is described in the HINTS methodology report (11). Briefly, H5c3 employed a two-stage, stratified random sampling methodology, first selecting residential addresses across the United States and then, one adult within each household. The database of residential addresses was classified into “high-minority strata” (areas with ≥34% Hispanics or African Americans) and low-minority strata (areas with <34% Hispanics or African Americans). Written informed consent was obtained from study participants. H5c3 was approved by the Westat Institutional Review Board and classified as exempt from review by the U.S. National Institutes of Health Office of Human Subjects Research Protections because the data were deidentified.

We examined the association between the use of EWDs and reported adherence of participants to nationally recommended levels of (i) general physical activity (≥150 min/week), (ii) resistance and strength exercise (≥2 times/week), and (iii) intentions to lose weight (Yes/No).

For the purposes of this study, individuals with depression and anxiety were determined using the answer of the participant to the question, “*Has a doctor or other health professional ever told you that you had depression or anxiety disorder (yes/no)?”*

The use of EWDs was self-reported and based on the responses of participants to the question “*In the past 12 months, have you used an electronic wearable device to monitor or track your health or activity? For example, a Fitbit, Apple Watch, or Garmin Vivofit?” Response options include Yes/No*. Other variables of interest include age, race/ethnicity, marital status, education level, gender, household income, health insurance status, self-rated health, access to a regular provider, number of comorbidities, and confidence in taking care of self. Replicate weights, based on the jackknife replication method (12), were applied to obtain accurate variance estimates.

The current study assessed the association between the use of EWDs and physical activity parameters using multivariable logistic regression and adjusted for all covariates depicted in Table 1. Our inclusion of covariates was informed by findings from previous research (13). All analyses were performed using Stata version 14.0 (StataCorp LLC, College Station, TX, USA). For all analyses, a two-tailed alpha level of 0.05 was considered statistically significant. We accounted for the complex survey design of the HINTS to ensure that our results are generalizable to the population in the United States.

**Table 1:** Sample population demographic characteristics: sample *n* = 1,139.

**Demographic variables**	**Total (*n* = 1,129), %^**a**^**	**Non-Use of EWDs (*n* = 862), %**	**Use of EWDs (*n* = 267), %**	**Test-Statistic**	***p-*value**
**Gender**				5.12	0.028
Female	60.99	67.36	32.64		
Male	39.01	77.69	22.31		
**Marital status**					
Not Married	54.33	71.89	28.11	0.04	0.838
Married	45.67	72.76	27.24		
**Age group**					
18–34	28.24	56.64	43.36	10.14	<0.001
35–49	26.45	70.67	29.33		
50–64	32.83	78.59	21.41		
65+	12.48	90.17	9.83		
**Education**					
Less than college	38.80	87.19	12.81	10.46	<0.001
Some college	33.78	66.13	33.87		
College graduate	16.09	59.10	40.90		
Post-graduate	11.33	58.30	41.70		
**Household income**					
< $20,000	26.24	83.63	16.37	5.29	<0.001
$20,000–$34,999	11.60	88.20	11.80		
$35,000–$49,999	14.21	65.65	34.35		
$50,000–$74,999	16.85	70.57	29.43		
$75,000 or more	31.10	58.25	41.75		
**Race**					
White	72.12	71.96	28.04	0.62	0.585
African American	8.35	77.93	22.07		
Hispanic	14.01	67.14	32.86		
Others	5.52	80.97	19.03		
**Insurance status**					
Not insured	7.33	76.08	23.92	0.17	0.682
Insured	92.67	71.46	28.54		
**Self-health status**					
Fair or Poor	27.36	83.98	16.02	8.19	<0.001
Good	39.04	72.46	27.54		
Excellent or Very Good	33.60	60.52	39.48		
**Confidence in taking care of own health**					
Somewhat or none	44.07	74.74	22.26	4.76	0.034
Completely or Very	55.93	67.05	32.95		
**Having a regular provider**					
No	26.34	83.63	16.37	1.27	0.266
Yes	73.66	87.78	12.22		
**Comorbidities**					
None	43.76	68.04	31.96	2.11	0.152
One or more	56.24	74.92	25.08		
**Physical activity**					
<150 min/week	71.14	76.46	23.54	10.60	0.002
>150 min/week	28.86	60.65	39.35		
**Resistance/strength exercise**					
No	66.91	75.85	24.15	5.47	0.023
Yes	33.09	63.98	36.02		
**Lose weight**					
No	45.89	80.57	19.43	6.05	0.018
Yes	54.11	64.80	35.20		

*n = sample size; Sample size may not add to total population (N = 1,139) due to missing responses*.

## Results

The sample distribution of the study population is depicted in Table 1. Overall, approximately one-third (28.6%) of the participants reported adherence to nationally recommended levels of general physical activity (≥150 min/week), 33% reported adherence to resistance and strength exercise (≥2 times/week), and more than half (54.1%) reported intention to lose weight in the last year. A total of 261 individuals (weighted percentage 28.1%) endorsed using EWDs in the last year.

In bivariate analysis (Table 1), individuals with depression and anxiety who reported using EWDs in the last year were more likely to be women (32.64%) vs. men (22.31%), college graduates (40.9%) vs. people with less than a college education (12.81%), people with high income (41.75% were from households with annual income ≥US$75,000) vs. people with lower income (16.37% were from households with annual income ≤ US $20,000), and people with excellent or very good self-rated health status (39.48%) vs. people with good or fair (27.54%) or poor self-rated health status (28.41%). Those with depression and anxiety who reported meeting the national recommendation for weekly physical activity (39.35 vs. 23.54%) and resistance or strength exercise training (36.02 vs. 24.15%) were more likely to have reported the use of EWDs than those who did not. Regarding race, 32.86% of Hispanics, 28.04% of Whites, and 22.07% of Blacks reported the use of EWD in the last year.

In the unadjusted models (Table 2), the use of EWDs was significantly associated with higher odds for meeting national recommendation for weekly physical activity (2.11; 95% *CI* 1.32–3.37; *p* = 0.002), and resistance/strength exercise (1.77, 95% *CI* 1.08 – 2.91; *p* = 0.026), as well as intention to lose weight in the last year (2.25; 95% *CI* 1.14–4.46; *p* = 0.021). However, after adjustment, the use of EWDs was only significantly associated with a higher odd of reporting intention to lose weight (2.12; 95% *CI* 1.04, 4.35; *p* = 0.04) (as shown in Table 2).

**Table 2:** Adjusted and unadjusted odds of association between electronic wearable devices (EWDs) use and physical activity parameters among individuals with depression and anxiety.

	**Unadjusted OR, 95% CI**	***p*-value**	**Adjusted OR, 95% CI**	***p*-value**
Physical activity	2.11 (1.32, 3.37)	0.002	1.65 (0.96, 2.85)	0.069
Strength/exercise training	1.77 (1.08, 2.91)	0.026	1.50 (0.76, 2.99)	0.239
Intention to lose weight	2.25 (1.14, 4.46)	0.021	2.12 (1.04, 4.35)	0.040

All the adjusted models adjusted for age, race/ethnicity, marital status, education level, gender, household income, health insurance status, self-rated health, access to a regular provider, number of comorbidities, and confidence in taking care of self.

OR: odds ratio; CI: confidence intervals.

## Discussion

In this study, we sought to explore how the use of EWDs may be associated with physical activity in people with mental illness. Specifically, we examined the prevalence of the use of EWDs and its association with general physical activity, resistance exercise, and weight loss intent among 1,139 individuals with self-reported diagnosis of depression and anxiety, from a nationally representative sample of the adults in the United States. The current study findings that 28.1% of those with depression and anxiety reported using wearable devices is similar to rates (28.2%) reported in previous research using the HINTS data (13) but also exceeds rates (21%) reported in the general population from the PEW Research Center (14). Thus, the present result supports past research (8) and demonstrates that these tools are acceptable by those with mental health conditions in tracking their health and highlights an opportunity to effectively utilize EWDs for continuous patient engagement.

Whereas, other studies have suggested that EWDs may result in increased physical activity levels among those with mental illness and in the general population (9, 13, 15), our results contrast those prior findings. We observed that in the bivariate analysis, the use of EWDs was significantly associated with higher odds for meeting national recommendations for weekly physical activity (OR 2.11), weekly resistance/strength exercise training (OR 1.77), and reporting intentions to lose weight in the last year (OR 2.25). After adjusting for potential sociodemographic and health-related confounders, the associations with meeting national recommendation for weekly physical activity and strength training are attenuated, and no longer statistically significant. However, after adjusting for potential sociodemographic and health related confounders, the use of EWDs was only associated with weight loss intentions in the last year, as respondents who used EWD had 2.12 times higher odds of reporting their intention to lose weight when compared with their counterparts not using EWDs.

Our observation that the use of EWDs is associated with intent to lose weight but not actual physical activity among people with depression and anxiety after controlling for confounding factors is novel and offers useful clinical insights on how to utilize these technologies to encourage physical activity. EWDs may serve as an opening for clinical interactions around physical health through identifying patients primed for behavior change. Beyond the setting of a clinic or face-face interaction, EWDs may be harnessed as a means to not just monitor physical activity but as a facilitator to provide personalized feedback interventions based on goal of the user and resultantly accelerate positive behavioral change.

Previous studies have documented the potential for using EWDs in continuous patient symptom monitoring, longitudinal data collection, supportive self-care, and early detection of relapse among people with mental illness (16). However, the role of EWDs in stimulating physical activity in those with mental illness remains to be proven. The results of the current study raise the possibility that the associations between EWDs and physical activity in mentally ill populations may be driven by confounding factors rather than directly linked. Therefore, the use of EWDs alone may not lead to improved activity and health among mentally ill populations. Rather, those with a mental disorder may require more customized support to achieve physical fitness. However, EWDs may certainly be used within physical activity interventions to augment traditional care.

The findings of this study should be examined within the context of some limitations. First, the cross-sectional nature of the study precludes our ability to draw causal conclusions between the use of EWDs and physical activity parameters. Second, the use of self-report measures to obtain physical activity levels could induce some level of social desirability or recall bias (17). Third, our study sample only represents those with depression or anxiety and not a broader population with mental illness. Fourth, we were limited by the sets of questions contained in the survey, and thus, further specific information on the type, characteristics, and purpose of using EWDs which may have influenced our findings could not be obtained. For example, it is possible that the EWD of a respondent is primarily used to assist them with health behaviors other than physical activity, such as sleep or dieting. Finally, it was not possible to ascertain the current status of the diagnosis and whether those who responded affirmatively to depression or anxiety had already received treatment and were no longer impacted by their illness. Therefore, future studies that utilize longitudinal designs, integrate objective measures of physical activity, such as accelerometers, and accounts for EWD features may offer useful insights into the role of EWDs in promoting physical health among those with mental health conditions.

## Conclusion

About three in 10 adults in the United States with depression and anxiety reported using EWDs in the last year. The current study findings suggest that among people with mental health conditions, the use of EWD is associated with higher odds of weight loss intent, but not with meeting the national recommendation for weekly physical activity or exercise/strength training. Further large-scale studies using randomized trial designs are needed to examine the causal relationships between EWDs and physical activity in people with mental health conditions, and to determine if what formats of additional support could be used alongside EWDs (such as text messaging, in-person training, or physical activity apps) to constitute an effective intervention for improving physical activity and health outcomes among people living with mental illness.

## Data Availability Statement

Publicly available datasets were analyzed in this study. This data can be found here: The data that support the findings of this study are openly available in [HINTS Public Use Dataset] at [https://hints.cancer.gov/data/download-data.aspx]. This data is publicly available and downloadable from the HINTS website.

## Ethics Statement

Ethical review and approval was not required for the study on human participants in accordance with the local legislation and institutional requirements. The patients/participants provided their written informed consent to participate in this study.

## Author Contributions

HO: conceptualization, data cleaning, data analysis, and writing. JF: conceptualization, writing, and editing. VE and CM: literature review, writing, and editing. JN and PB: writing and editing. All authors contributed to the article and approved the submitted version.

## Funding

JF is supported by a University of Manchester Presidential Fellowship (P123958) and a UK Research and Innovation Future Leaders Fellowship (MR/T021780/1) and has received honoraria / consultancy fees from Atheneum, ParachuteBH and Nirakara, independent of this work. JT is supported by an American Psychiatric Association Research Fellowship.

## Conflict of Interest

JF is supported by a University of Manchester Presidential Fellowship (P123958) and a UK Research and Innovation Future Leaders Fellowship (MR/T021780/1) and has received honoraria/consultancy fees from Atheneum, ParachuteBH and Nirakara, independent of this work. JT is supported by an American Psychiatric Association Research Fellowship. The remaining authors declare that the research was conducted in the absence of any commercial or financial relationships that could be construed as a potential conflict of interest.

## Publisher's Note

All claims expressed in this article are solely those of the authors and do not necessarily represent those of their affiliated organizations, or those of the publisher, the editors and the reviewers. Any product that may be evaluated in this article, or claim that may be made by its manufacturer, is not guaranteed or endorsed by the publisher.
